# Spatio-Temporal Trends in Wildlife-Vehicle Collisions: Implications for Socio-Ecological Sustainability

**DOI:** 10.3390/ani15101478

**Published:** 2025-05-20

**Authors:** Manju Shree Thakur, Prakash Chandra Aryal, Hari Prasad Pandey, Tek Narayan Maraseni

**Affiliations:** 1 Faculty of Environmental Science, GoldenGate International College, Kathmandu 44600, Nepal; mshree342@gmail.com (M.S.T.); arc.prakash@gmail.com (P.C.A.); 2 Environment Protection and Study Center, Kathmandu 44600, Nepal; 3 Institute of Life Sciences and the Environment, University of Southern Queensland, Toowoomba, QLD 4350, Australia; tek.maraseni@usq.edu.au; 4 Ministry of Forests and Environment, Government of Nepal, Kathmandu 44600, Nepal; 5 The Northwest Institute of Eco-Environment and Resources, Chinese Academy of Sciences, Lanzhou 730000, China

**Keywords:** co-existence, roadkill, socio-ecology, tiger prey-base, wildlife-vehicle collision

## Abstract

The conservation of biodiversity and the balance between ecological and societal needs are critical but often contested global issues. Wildlife-vehicle collision (WVC) on vital infrastructure, especially linear infrastructure, remains a persistent challenge from policy to practice and poses a serious life-threatening implication to humans and other non-human lives. Addressing this issue effectively requires solutions that provide win-win outcomes from both ecological and societal perspectives. This study critically analyzes a decade of roadkill incidents along Nepal’s longest East-West national highway, which passes through a biologically diverse national park in the western Terai Arc Landscape Area (TAL). Findings are drawn from field-based primary data collection of the period 2012–2022, secondary literature review, key informant interviews, and spatial analysis. The study reveals significant variations in roadkill incidence across areas and years. Despite Bardia National Park being larger and having a higher wildlife density, Banke National Park recorded higher roadkill rates. This is attributed to insufficient mitigation measures and law enforcement, more straight highway segments, and the absence of buffer zones between the core park and adjacent forest areas—only a road separates them. Wild boars (*Sus scrofa*) and spotted deer (*Axis axis*), the primary prey of Bengal tigers (*Panthera tigris tigris*), were the most frequently road-killed species. This may contribute to human-tiger conflicts, as observed in the study areas. Seasonal trends showed that reptiles were at higher risk during the wet season and mammals during winter. Hotspots were often located near checkpoints and water bodies, highlighting the need for targeted mitigation efforts such as wildlife crossings and provisioning wildlife requirements such as water, grassland, and shelter away from the regular traffic roads. Roadkill frequency was also influenced by forest cover and time of day, with more incidents occurring at dawn and dusk when most of the herbivores become more active in search of food, shelter, water, and their herds. The findings underscore the importance of road characteristics, animal behavior, and landscape features in roadkill occurrences. Effective mitigation strategies include wildlife crossings, speed limits, warning signs, and public education campaigns. Further research is needed to understand the factors in driving variations between parks and to assess the effectiveness of mitigation measures.

## Simple Summary

Wildlife getting hit by vehicles on highways is a big problem, especially in areas where roads pass through forests and national parks. This not only harms animals but can also be dangerous for people. A study looked at 10 years of animal deaths along a major highway in Nepal that cuts through a wildlife-rich area. It found that more animals were killed in Banke National Park than in nearby Bardia National Park. This was likely because Bardia has better rules and safety measures in place, while Banke has more straight roads and disturbed areas. The animals most often hit were wild boars and spotted deer—key prey for tigers—which may cause tigers to move closer to villages in search of food. The study also showed that reptiles were more at risk in the rainy season, while mammals were hit more in winter. Most accidents happen near water sources and at dawn or dusk when animals are active. Our study suggests adding wildlife crossings, placing food and water away from roads, and installing signs or speed limits in risky areas. Results indicate the need for better planning and understanding of animal behavior to make roads safer for all.

## 1. Introduction

Balancing infrastructure development with ecosystem conservation remains a global challenge, especially in terrestrial environments where road connectivity is in high demand. While road transport dominates land transportation [1], it significantly impacts wildlife behavior and movement. Poorly planned roads can lead to accidents involving people and animals, forest fragmentation, and biodiversity loss [2,3]. Unplanned linear infrastructure such as roads threatens biodiversity by physically fragmenting habitats [4] and indirectly by degrading landscape quality [5]. They contribute to wildlife mortality [6] and disrupt continuous habitats, reducing their quantity and quality while creating edge effects [7,8,9,10,11]. To balance infrastructure needs with ecological preservation, it is crucial to integrate ground realities into policy decisions, ensuring sustainable development that benefits both society and biodiversity.

Wildlife-vehicle collisions (WVC) are a growing global issue, fragmenting wildlife populations and making humans more vulnerable. Roads disrupt the movement and distribution of various species, including mammals, birds, reptiles, and amphibians [12,13,14]. These collisions not only reduce wildlife numbers and disrupt ecological functions but also pose serious risks to human safety, often leading to accidents and fatalities. Human impacts on faunal populations are evident worldwide, with roadkill—a lethal interaction between wildlife and vehicles [15]—commonly reported in urban, rural, and wild regions [16]. WVC significantly affects ecological populations and communities [17,18,19], increasing threats to biodiversity [20]. The environmental impacts of WVC include restricted wildlife movement, altered behavior, migration disruptions, and habitat fragmentation, leading to functional isolation of populations. The severity of WVC depends on multiple factors, including vehicle speed, road type, wildlife density, visibility, driver awareness, and seasonal variations [21]. Road width, proximity to forests [22,23], animal movement patterns, landscape features, and road characteristics all influence the likelihood of wildlife-vehicle collisions (WVC) [24,25]. Additional factors such as vehicle type, driving speed, habitat needs, visibility, weather, and daily wildlife activity patterns may also affect the frequency and severity of WVC, though these are often underreported [13,14].

Past studies show that roadkill happens more often in places with thick plants near the road, which makes it hard for drivers to see [26,27,28], and among animals that move around a lot or get easily scared by traffic [28,29]. Road fatalities increase with higher traffic speeds, especially on paved roads [26], though heavy traffic can act as a barrier to wildlife [2]. Despite lower nighttime traffic, nocturnal and crepuscular species face higher risks due to reduced driver visibility [30]. However, in the Indian sub-continent, such as in Nepal, these patterns differ due to high prey and predator densities in national parks, heavy forest dependence among local communities, habitat encroachment, poor road and vehicle conditions, inadequate wildlife crossings, and limited signage and surveillance. A report by the Department of National Parks and Wildlife Conservation [31] states that nearly 300 animals were killed by vehicles in protected areas within a fiscal year. Wildlife-vehicle conflicts are most frequent in Nepal’s Terai National Parks, particularly in Banke and Bardia, with Banke recording the highest number of fatalities [31]. However, the government has neither identified the root causes nor has research been conducted to analyze contributing factors [32,33]. The issues of roadkill and biodiversity loss could extend to mountainous areas as roads are gradually expanding there as well. A recent study highlighted a correlation between physical offsets and wildlife collisions [34]. Other than natural deaths, roadkill is the main reason for wildlife deaths in an unnatural way, followed by dog attacks, poaching, and others [29]. Addressing these gaps is crucial for safeguarding shared social-ecological landscapes and informing policy revisions for effective mitigation [35,36]. To address the challenges of genetic isolation and biodiversity loss [27] while ensuring road safety [32,37,38], this study examines key gaps in the literature through a case study of two adjacent national parks in Nepal, traversed by the country’s longest highway.

This study endeavors to understand the status and implications of roadkill in two lowland national parks of Nepal. Specifically, we seek to: (1) assess roadkill occurrences in Banke and Bardia NPs along the East-West (then Mahendra highway) national highway, (2) analyze spatial and seasonal roadkill trends, and (3) identify contributing factors at both sites and explore potential policy solutions. The findings provide insights and information into how road features, animal behavior, and landscapes influence roadkill for sustainable planning. Mitigation like crossings, speed limits, and awareness can help, but more research is needed to understand park differences and strategy effectiveness. As Nepal’s participatory biodiversity conservation efforts serve as a model [39], the findings offer insights to inform national and global decision-making, promoting socio-ecological sustainability [36,39,40] and coexistence on our shared planet for environmental justice [41,42].

## 2. Methods

### 2.1. Study Area

**Figure 1:**
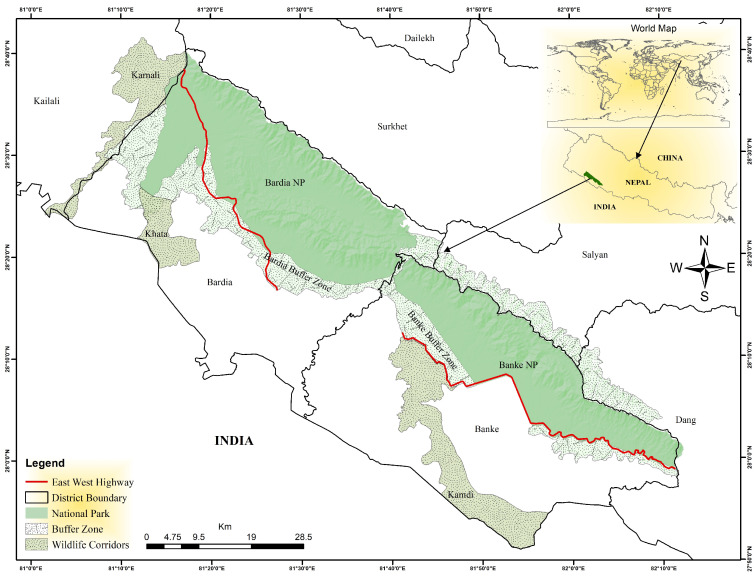
A study area map depicting the Highway, both national parks, their buffer zones, hillsides, and corridors. Refer to the legend for details.

The study was conducted in Banke National Park (BaNP) and Bardia National Park (BNP) in Nepal’s mid-western lowlands (Figure 1). BaNP, established in 2010, spans 550 km^2^ with 343 km^2^ buffer zone and is located between 81°39′29″ to 82°12′19″ E and 27°58′13″ to 28°21′26″ N [43]. BNP, established in 1976, covers 968 km^2^ with 327 km^2^ buffer zone and lies between 28°40′14.5″ to 28°17′13.4″ N and 81°12′8.838″ to 81°42′55″ E [44]. Nepal’s busiest road, the East-West Highway, passes through these protected areas (Figure 1). BaNP is connected to BNP and is connected to India’s Katerniaghat Wildlife Sanctuary via the Kamdi Corridor whereas Bardia NP connects to India via Khata Corridor, wilderness areas, and community forests.

Banke NP is home to 124 plant species, 34 mammal species, over 300 bird species, 24 reptile species, 7 amphibian species, and 58 fish species. *Shorea robusta*, *Acacia catechu*, and *Dalbergia sissoo* constitute the majority (90%) of the natural forest cover [45]. The elevation of Banke NP ranges between 153 m and 1247 m [43]. Similarly, Bardia NP hosts more than 30 mammal species, 230 bird species, and numerous reptiles and fish species. The natural forest cover in BNP is primarily composed of *Shorea robusta*, *Neolamarckia cadamba*, *Dalbergia sissoo*, *Bombax ceiba*, and *Syzygium cumini*. Its elevation ranges from 152 m to 1441 m [44,45].

Both parks experience temperatures ranging from a minimum of 10 °C to a maximum of 45 °C, with January being the coolest month, May–June the hottest, July the wettest, and November the driest [46,47]. Although seasonal classifications vary across reports for these regions, in this study we define the seasons as follows: spring/pre-monsoon (March, April, May); summer monsoon (June, July, August, September); autumn/post-monsoon (October, November); and winter (December, January, February) [32,37,38]. Both national parks are home to indigenous and local communities, such as the Tharu people, whose livelihoods often depend on natural resources, eco-tourism, and traditional agricultural practices. Many communities living in the buffer zones surrounding the park rely on natural resources for their livelihoods, including timber, non-timber forest products, and agriculture.

East-West Highway NH58 (72 km) and NH59 (50 km) fall within our study area Banke and Bardia National Parks respectively. These highways currently have two lanes, each 6–7 m in width, with a proposed expansion to 24 m (four lanes) in the forest area. Traffic data for Kohalpur East, representing Banke NP, and Kohalpur West, representing Bardia NP, were obtained from the Department of Roads, Nepal [48].

Several small rivers and rivulets drain the area. The main rivers in the region are the Karnali, Rapti, and Babai Rivers, and their key tributaries, including Shiva Khola, Sukhar Khola, Rohini Khola, and Khairi Khola, cross the road (east-west) from north to south. Most of the hills in the area are in the northern parts and are oriented southward, and most of the region’s territory consists of flat areas.

### 2.2. Data Collection and Analysis

Since all roadkill data for protected areas (PAs) are managed and maintained by the PAs’ authorities and the Department of National Parks and Wildlife Conservation (DNPWC), we obtained raw data from the Banke NP and Bardia NP authorities for this study. We categorized the vertebrate taxonomy based on the provided data for the years 2015 to 2022 for Banke NP and 2012 to 2022 (except for 2014 and 2015, for which data are missing) for Bardia NP, following the IUCN Red List and Jnawali et al. (2011); for unknown species, it is categorized as a common name and listed as not assessed (NA) [49,50]. We tested whether roadkill incidents occurred randomly or if they were spatially clustered for the available georeferenced location. Roadkill sites for both PAs were mapped as hotspots using the Kernel density tool in ArcGIS 10.4.1, based on the GPS locations of the roadkill within the selected time frame.

The 72 km-long road in BaNP was divided into 72 sections, each 1 km in length. Similarly, the 50 km road through BNP was divided into 50 sections of 1 km each [43]. Each section had a 1 km buffer zone, creating a 1:1 ratio for the road segments to the buffer zone. Buffer zones with available roadkill GPS points and Land Use Land Cover (LULC) data for 2017 and 2020 were used, sourced from Esri Sentinel-2 imagery (Version 003) with 10 m resolution. The image data were downloaded, unzipped, and added to ArcGIS. The data were clipped to match the buffer segments, and raster data were converted into polygons. The “Identify” tool was used to determine land cover types for each buffer zone and to calculate the area for each variable. These variables included water, forest, grassland, bare land, and cropland. Area values were calculated in ArcGIS and exported to Excel, along with the number of roadkill points per year, for further analysis. Additionally, highway curves were manually counted for each road segment. Curves were identified in ArcGIS by visualizing turn angles and turns greater than 45° (ranging from soft to sharp) were categorized as curves in this study.

Similarly, for hotspot analysis, the spatial distribution of wildlife accidents along the highway was analyzed using ArcGIS version 10.4.1.The Kernel Density Estimation (KDE) [14,36] was applied, and Moran’s Index [13] was calculated on the KDE output values to determine spatial autocorrelation.

Moreover, data analysis involved regression techniques and modeling. Inferential statistics, such as Wilcoxon signed rank tests, were used to compare the roadkill between two PAs and between years. The roadkill per 1 km of the road section (representing the land area 1 km by 1 km square grids) was modeled using a Poisson error structure using land cover and land use variables and road turnings as predictor variables. This included water, forest, grassland, bare land, cropland, and turning roads. The data analysis involved data management in spreadsheets and statistical analysis using R [51] and Rstudio [52], employing its various libraries such as mgcv [53], ggplot2 [54].

To check for the seasonality of the roadkill events, we modeled the roadkill data and monthly records from 2016 to the end of 2022 using a generalized additive model. We built the model with monthly roadkill records as responses and year and month as smooth predictor variables, where monthly variations to capture the seasonality in the data were included. We used a cyclic cubic spine with a value of 10 to capture monthly variations in the roadkill and built the model with a negative binomial structure, as this was better compared to the Poisson model (dispersion = 1.2).

## 3. Results

### 3.1. Roadkill Incidents in the Study Area

**Figure 2:**
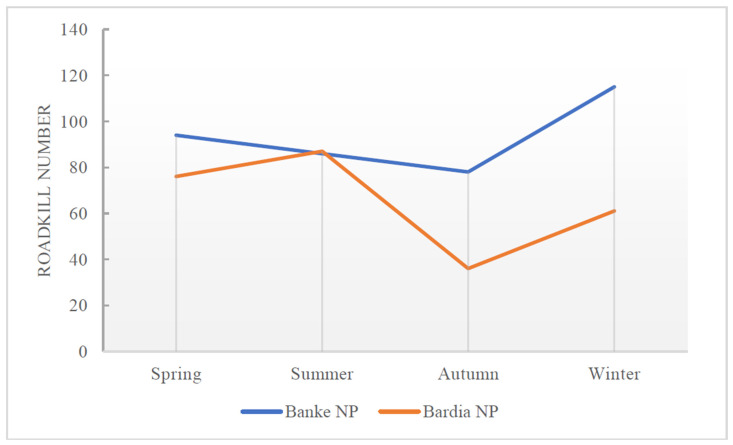
Seasonal pattern of roadkill in Banke NP (Blue) and Bardia NP (Orange) over the years (2016–2022).

**Figure A1:**
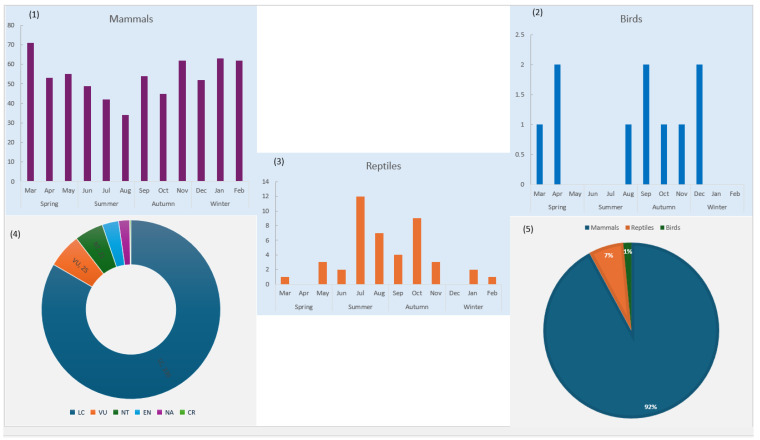
Order-wise wildlife roadkill in both Banke and Bardia National Parks during the study period: (**1**) seasonal and monthly distribution of mammal roadkill, (**2**) seasonal and monthly distribution of bird roadkill, (**3**) seasonal and monthly distribution of reptile roadkill, (**4**) wildlife roadkill by IUCN Red List category, and (**5**) proportion of roadkill by taxonomic order.

During the years 2016–2022, the annual average number of roadkill reported in Banke NP was 51.83, while the average in Bardia NP was 38.67. In Banke NP, the highest number of roadkill was recorded in 2017 *(n* = 70), and the lowest in 2020 (*n* = 35). In Bardia NP, the highest number of roadkill occurred in 2016 (*n* = 50) and 2020 (*n* = 49), while the lowest was in 2018 (*n* = 32). The mean difference in roadkill counts between the two parks (13, CI = −5.74 to 32.07, *p* = 0.133) is not significant. In comparing the seasonal variations in roadkill, Banke NP experienced a higher number of roadkill compared to Bardia NP, however, it experienced almost equal numbers of roadkill in the summer season (Figure 2). Out of the total roadkill species in both parks for all years, 91% were mammal groups followed by 8% of reptiles and birds at only 1% (Appendix A.1, Figure A1(1–3,5). Most species killed in road accidents are listed as Least Concern (LC), followed by Vulnerable (VU), Near Threatened (NT), Endangered (EN), unknown (NA) status, and Critically Endangered (CR) (Appendix A.1, Figure A1(4)).

**Figure 3:**
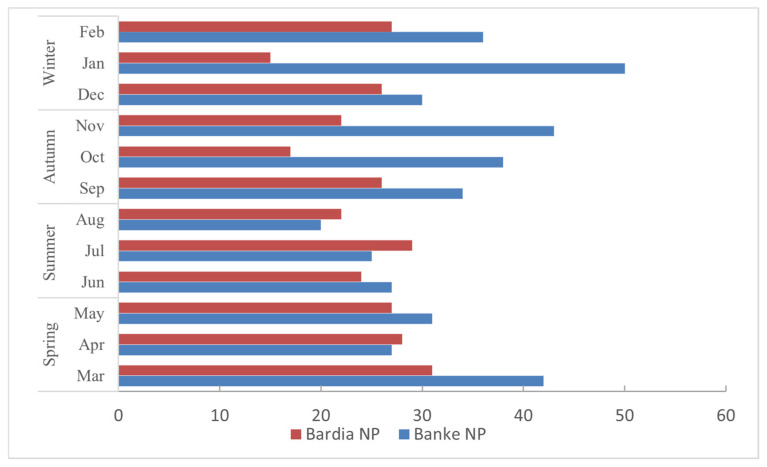
Monthly roadkill records for the two national parks.

**Figure 4:**
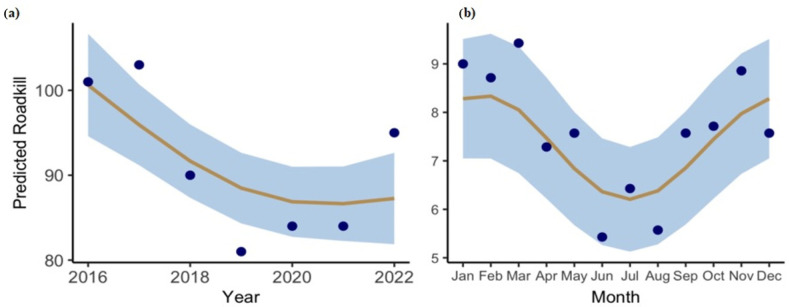
Predicted roadkill for years (**a**) and months (**b**). The points are predicted roadkill, the lines are the predicted regression trend, and the shaded regions are the confidence intervals.

Monthly roadkill data show (Figure 3) notable variations for Banke NP (range = 18–50, mean = 31.08, SD = 9.71) compared to Bardia NP (range =13–27, mean = 21.67, SD = 4.27), with a significant difference in the roadkill between the two parks (*p* = 0.019). Banke National Park (BaNP) records predicted a higher number of roadkill compared to Bardia NP every year except 2020 (Figure 4). Although seasonal variations are seen, the highest roadkill numbers were seen during winter in BaNP and during summer in BNP; fewer accidents were seen in Autumn in both parks.

The predicted roadkill for the data years showed a variation and a decline in accidents (Figure 4a), but the decline was non-significant. However, there was a significant monthly variation (Table 1) with a strong seasonality effect (Figure 4b) in the roadkill.

The highly significant (β = 2.02, z = 45.64, *p* < 2 × 10^−16^) intercept, the model estimate of around 8 roadkill, shows a strong baseline effect for predictors at their reference values. Since the dispersion parameter for the model was found to be 32.12, we can see the justification for the model. Moreover, the month looks more important (χ^2^ = 7.73, edf = 1.94, *p* = 0.008) than the non-significant effects of the year (χ^2^= 1.25, edf = 1.45, *p* = 0.36).

**Figure A2:**
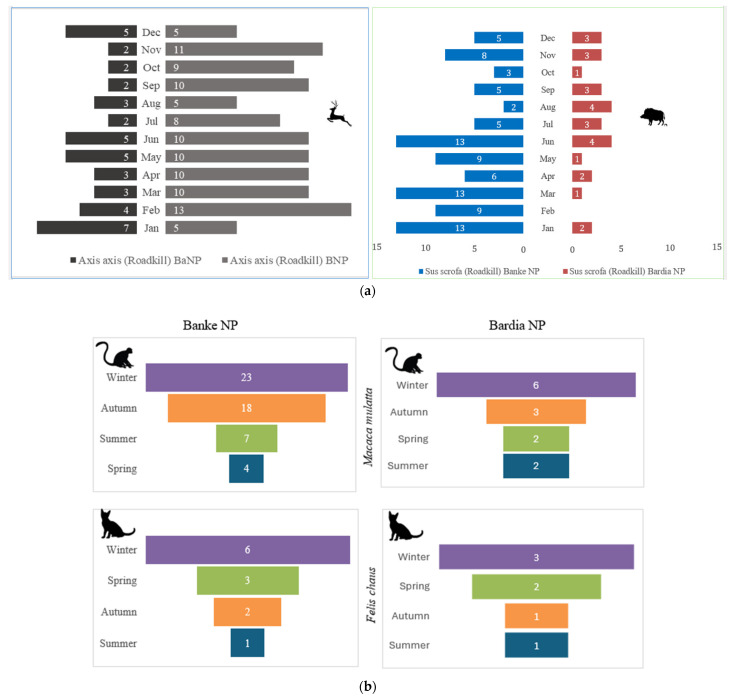

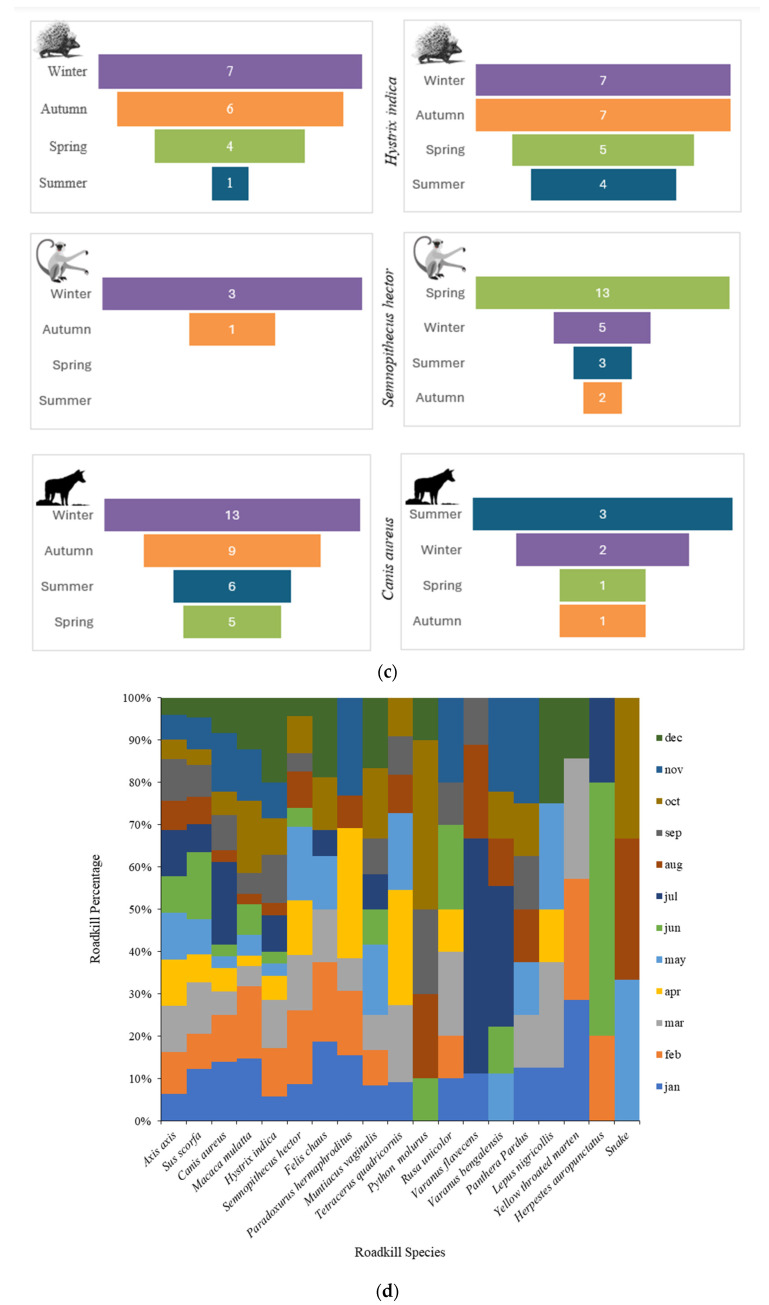
(**a**) The *Axis axis* and *Sus scrofa* roadkill in both national parks. (**b**) The *Macaca mulatta* and *Felis chaus* roadkill in both national parks. (**c**) The *Hystrix indica*, *Semnopithecus hector* and *Canis aureus* seasonal patterns of roadkill in both parks. (**d**) Graphs showing the species roadkill on a month-by-month basis.

Out of 695 total roadkill events recorded, 36 species were killed in Banke NP and 24 species in Bardia NP. It was found that *Axis axis* roadkill occurred year-round, with the highest number of accidents between February and July (see details in Appendix A.2, Figure A2a, left). We observed a significant difference in the number of *Axis axis* roadkill between Bardia NP and Banke NP (V = 76.5, *p* = 0.0032). Similarly, *Sus scrofa* is the second most killed species in vehicle collisions, with most of the accidents occurring in January, March, and July (see details in Appendix A.2, Figure A2a, right). Banke NP has a significantly higher overall roadkill of *Sus scrofa* compared to Bardia NP (*p* = 0.006), and seasonal variations are more noticeable in Banke NP than in Bardia NP. The third most killed species is *Canis aureus*, mostly killed in July, followed by January, November, and February. The fourth most frequently killed species is *Macaca mulatta*, with most accidents occurring in February, October, January, November, and December. *Paradoxurus hermaphroditus* is most killed in April and November, while *Python molurus* is primarily killed in October (see details in Appendix A.2, Figure A2c,d).

### 3.2. Hotspot Analysis of Roadkill in Banke and Bardia National Parks

**Figure 5:**
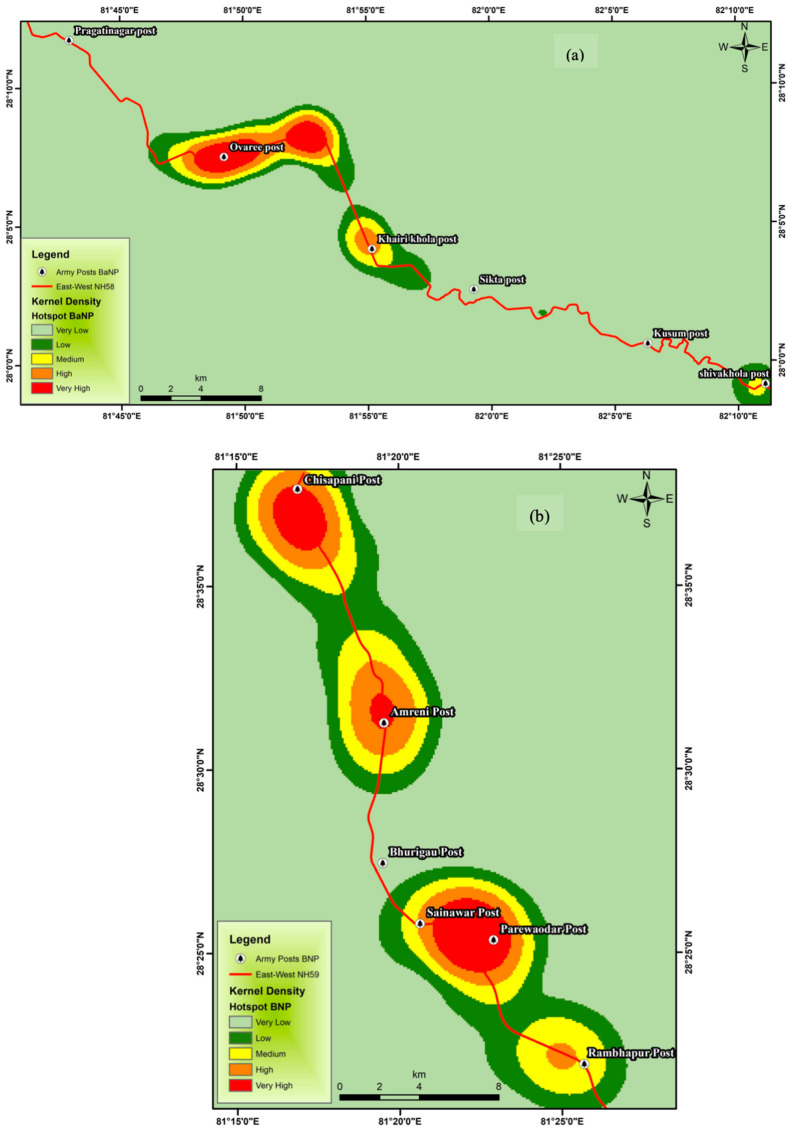
Identified hotspots of roadkill in Banke and Bardia NPs. Hotspots of roadkill in Banke National Park (**a**); hotspot of roadkill in Bardia National Park (**b**).

**Figure A3:**
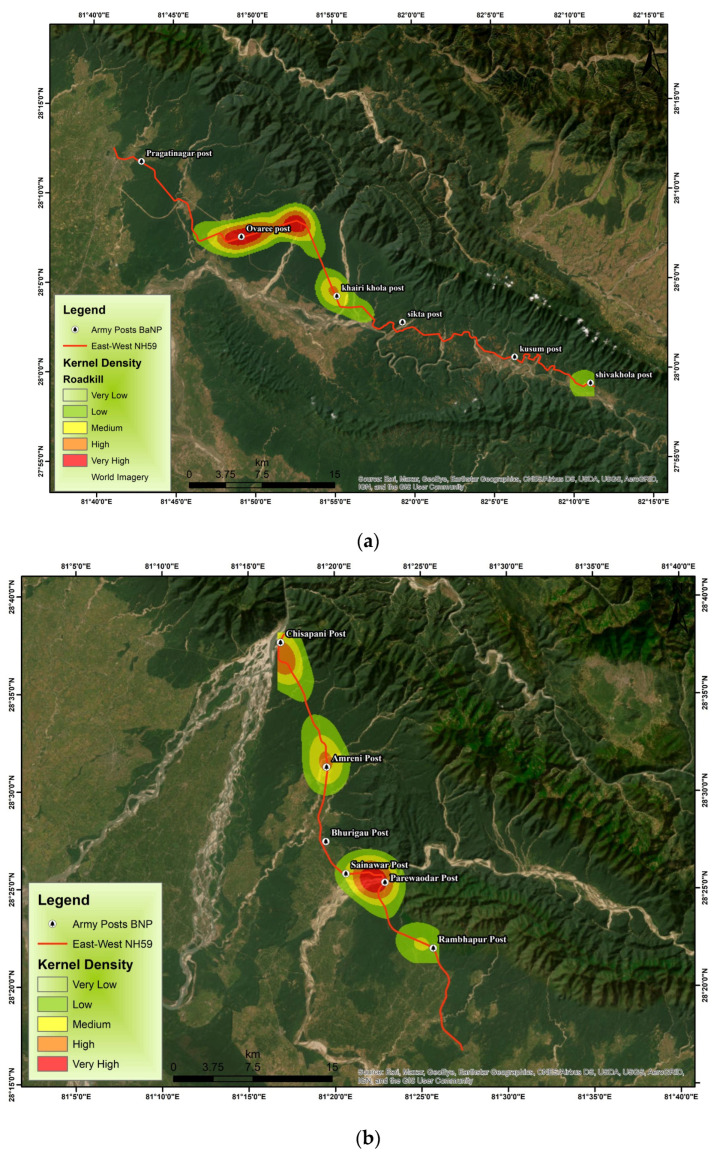
(**a**) Hotspot areas of Banke National Parks with check posts. (**b**) Hotspot areas of Bardia National Park with check posts.

Overall, the Ovaree post, Khairi Khola post, and Shiva Khola post were identified as the three main hotspots for roadkill in Banke NP (Figure 5a). Similarly, four major roadkill hotspots, namely, Rambhapur post, Parewaodar post, Amreni post, and Chisapani post in Bardia NP, were found (Figure 5b). The Moran’s Index (I = 0.219) with an expected value (−0.007463), significant spatial autocorrelation was observed (z = 7.498, *p* = 0.00, variance = 0.0008), reflecting that the roadkill is clustered in the hotspots (see further details on hotspot analysis in Appendix A.3, Figure A3a,b).

### 3.3. Relation with Predicted Variables of Roadkill

**Figure 6:**
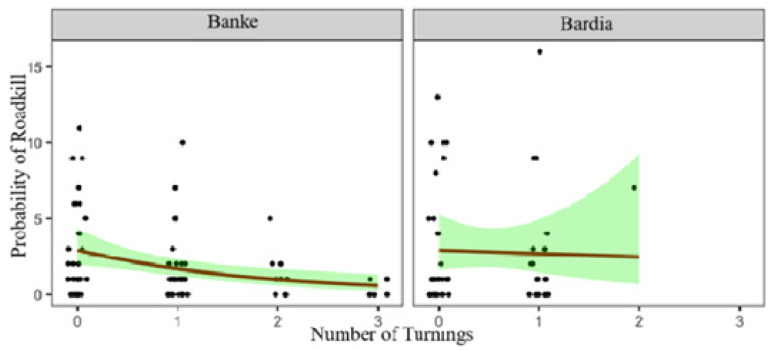
Relationship between roadkill and number of turns (road curvatures) for Banke and Bardia National Parks. The points represent the number of roadkill, lines represent the lines of best fit, and shaded regions represent confidence intervals.

**Figure 7:**
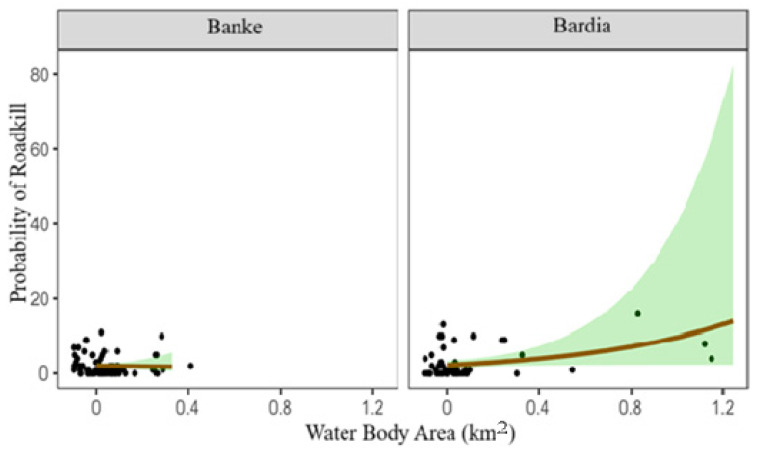
Relationship between roadkill and water body area. The points are the number of roadkill, lines represent the lines of best fit, and shaded regions represent confidence intervals for both National Parks.

While examining the relationship between roadkill and selected variables, it was found that the area of water bodies and the number of turnings significantly influence roadkill events (Table 2). Although not statistically significant, cropland areas tend to reduce roadkill, while forested areas increase roadkill. Regardless of the species killed, the area of water bodies significantly increases roadkill (β = 1.22, *p* = 0.04, CI = 0.05 to 2.71), while the number of road turns decreases roadkill (β = −0.39, *p* = 0.017, CI = −0.73 to −0.06). In Banke NP, roadkill probability decreases with more road turnings, showing a strong and consistent effect (Figure 6). In contrast, Bardia NP shows a slight, non-significant increase in roadkill near larger waterbodies, though wide confidence bands suggest caution in interpretation (Figure 7).

## 4. Discussion

The findings highlight the significant impact of roadkill on wildlife, with vehicle accidents contributing to 23% of wild animal deaths, which is significant among unnatural deaths in a protected area in 2020 [31]. The relatively lower amount of roadkill in 2020 was attributed to mobility restrictions during the COVID-19 pandemic. Banke National Park (BaNP) consistently recorded higher roadkill rates than Bardia National Park (BNP), with key hotspots identified near specific posts in both parks. Factors such as the presence of water bodies, road curvatures, traffic volumes, and climate variables like temperature and precipitation were found to influence roadkill incidents [34]. Mammals, especially wild boar and spotted deer, were most affected, leading to a potential disruption of predator-prey dynamics, particularly impacting the tiger population [55]. These findings underscore the urgent need for targeted mitigation measures, such as wildlife crossings, traffic management, and habitat protection, to reduce roadkill and safeguard both wildlife and human safety. Additionally, seasonal variations and specific hot spots call for tailored interventions that address the underlying causes, including traffic volume and climate factors, to ensure the long-term sustainability of socio-ecological landscapes [39,42].

The results of the study indicate that the incidence of roadkill varied significantly across the study areas during the examined years. Despite Bardia National Park having a larger area and higher wildlife density than Banke National Park during the same fiscal years [55], Banke recorded a higher overall incidence of roadkill. This may be due to factors such as longer highway stretches, lack of effective vehicle speed mitigation, insufficient patrolling, absence of buffer zones between the core and surrounding forests, and a greater presence of straight roads and disturbed areas in Banke compared to Bardia [28,33]. Previous studies have highlighted factors like human activities (e.g., road construction, urbanization) and climate change-induced behavioral changes in animals as influences on roadkill frequency and location [56]. Effective mitigation measures, such as wildlife crossings (underpasses and overpasses) widely used in North America and Europe, have reduced roadkill incidents [18,57]. Other methods, including warning signs [58], fencing [57], and speed limits [22], should be employed to reduce nighttime speed limits for effectiveness in preventing wildlife-vehicle collisions as observed in BNP in our case.

We found that the wild boar (*Sus scrofa*) is the most frequently road-killed species in both national parks, particularly in Banke NP. These omnivores consume a variety of plant and animal matter, including roots, bulbs, seeds, nuts, green plants, insects, mice, frogs, and more [59]. Despite being listed under the “Least Concern” category of the IUCN Red List [49] due to its sustainable population size, roadkill poses a significant threat to its population if mitigation efforts are not implemented to protect the major predators due to their prey base [55]. Tigers, wolves, and leopards are the species’ main predators [60]. On the other hand, according to the study, the most frequently road-killed herbivore in the national parks is the axis deer (*Axis axis*). As a keystone species, tigers prey on these herbivorous mammals [61]. Since most deer species are herd animals, their movements can be tracked, and their behavior can help anticipate a rise in the risk of roadkill [62,63]. Spotted deer, for instance, primarily browse vegetation during the dry season, but they also graze on short grasses in open meadows within moist semi-evergreen forests [64,65]. Compared to Banke NP, which is mostly covered by mixed forests, Bardia NP has more grasslands, making it a preferred habitat for herbivores like spotted deer [61].

We observed seasonal effects on roadkill incidents in Banke and Bardia National Parks, with reptiles being at higher risk during the wet season due to mating and weather conditions, and mammals being at greater risk during the winter season. This finding aligns with a study that found that reptiles are at higher risk during the wet season [22,66]. We also found that most roadkill hotspots were located near checkpoints and water bodies near the roads, suggesting a positive correlation between roadkill and proximity to water. This may be because wildlife is drawn to limited water sources, especially during the dry season (spring and summer). Higher roadkill near checkpoints may be due to increased observation and reporting by park authorities in these areas. In contrast, incidents in less monitored areas, such as the middle of the highway, may go unreported unless seen by patrolling teams or others. Additionally, some incidents may not be reported due to fear of legal consequences, which could create difficulties for drivers and passengers. This highlights how the reporting of incidents is influenced by the relationship between park authorities and local communities, as well as by legal practices in developing countries like Nepal. This finding is consistent with previous studies, which have shown that roads near or between forest sections are high-risk areas for wildlife-vehicle collisions [4,62].

Another important factor that can influence roadkill incidents is the time of day. Many species, such as deer and other ungulates, tend to be more active during dawn and dusk, periods when visibility is reduced due to low light conditions. This increase in activity during these times significantly raises their chances of colliding with vehicles [61,62]. Our findings support this observation, as roadkill incidents were notably higher during these periods in both Banke and Bardia National Parks. The reduced visibility, combined with animals’ natural behaviors, such as foraging or migrating during these times, makes them more vulnerable to road collisions [19]. This emphasizes the need to consider time-of-day patterns when developing strategies to mitigate roadkill, such as adjusting traffic flow or implementing animal crossing structures.

We observed that hotspots of roadkill were often located near water sources, such as Khairi Khola, Shiva Khola, and Muguwa Khola, among others. This finding is consistent with previous research, which suggests that water bodies are significant influencing factors in the presence and movement of wildlife in the dry seasons and regions [39,62]. Animals may be attracted to water sources and crossroads or move to different locations to access them, increasing their risk of being struck by vehicles [2]. Therefore, the presence of hotspots near water regions in our study emphasizes the need for targeted mitigation efforts in these areas, such as wildlife crossings or reduced speed limits as already in practice in Bardia National Park [44].

Several studies have also reported a correlation between roadkill hotspots and water bodies. For example, a study conducted in the Western Ghats of India found that roadkill hotspots were concentrated near water sources, which wildlife used for drinking and other activities [67]. Another study in the United States found that roadkill was more likely to occur near water bodies, possibly because these areas provided suitable habitat for wildlife and acted as barriers to movement [68]. In addition to water bodies, several other factors have been found to influence the occurrence of roadkill in our study area. These include road characteristics (such as speed limits, road width, and traffic volume), animal behavior (such as crossing behavior and activity patterns), and landscape features (such as vegetation cover and habitat fragmentation) [25], ecosystem dynamics, climate change and its impact on animal behaviors, park-people relationships [40,42], and environmental justice (i.e., distribution of environmental goods and bads among the people irrespective of their background) [41,42].

We noted that habitat fragmentation is a major concern for wildlife conservation, as it can lead to the isolation of populations and reduce genetic diversity, increasing their vulnerability to roadkill and other threats. A study conducted in Brazil found that roadkill hotspots were concentrated in areas with high levels of habitat fragmentation, suggesting that habitat connectivity plays a crucial role in reducing the risk of roadkill [69]. Overall, understanding the factors that contribute to roadkill is essential for developing effective mitigation measures and reducing the impact of roads on wildlife populations. By identifying hotspots and key influencing variables, conservationists and transportation planners can collaborate to implement strategies that minimize the risk of roadkill and enhance the connectivity of wildlife habitats in a participatory manner [39,61].

There could be several reasons for the difference in major wildlife roadkill between Banke National Park (BaNP) and Bardia National Park (BNP). One possible reason is the difference in habitat and food availability [34,55,70]. *Sus scrofa*, the most frequently road-killed species in BaNP, is an omnivore that feeds on a variety of food items, including roots, bulbs, seeds, nuts, green plants, insects, mice, and frogs. In contrast, *Axis axis*, the most frequently killed species in BNP, is an herbivore that primarily feeds on grasses, leaves, and shoots. Another possible reason is the difference in traffic density and road infrastructure between the two parks. BaNP has straighter highway stretches and disturbed areas covered by roadways, which may increase the likelihood of wildlife-vehicle collisions. In contrast, BNP has implemented several mitigation measures and rigorous laws, such as a timecard system for vehicles, which may help reduce the incidence of roadkill.

The differences in major roadkill species between Banke National Park (BaNP) and Bardia National Park (BNP) could be influenced by a combination of factors, including human activities, climate change, and habitat changes. Human activities like land use changes, agriculture, and urbanization can alter wildlife movement patterns, increasing road exposure. Climate change may also affect species’ behavior and distribution, making them more likely to crossroads in search of food or water. Habitat changes, such as forest fragmentation, further influence wildlife movements and collision risks [13,36]. These factors likely contribute to the differences in roadkill species between the two parks, with further research needed to fully understand these dynamics [34]. In terms of mitigation, specific measures can target the most affected species. For *Sus scrofa* in BaNP, establishing feeding stations and installing fencing or wildlife crossings could reduce roadkill [63]. For *Axis axis* in BNP, reflective markers or light-reflecting devices could alert deer to oncoming vehicles [8], and modifying road designs with curves and vegetation barriers may also help reduce collisions [9,63]; this, in turn, could reduce the human-tiger conflict in the regions [31,55].

Public education campaigns can also play a key role in reducing roadkill incidents by raising driver awareness about the importance of avoiding wildlife collisions [63]. It is crucial to implement and monitor the effectiveness of these mitigation measures to ensure they are properly targeted and are successful in reducing roadkill. Additionally, our findings indicate that forests significantly influence roadkill factors, as they can obstruct animals from drivers’ view. This aligns with a study by Clevenger et al. (2001), which found that wildlife-vehicle collisions are more likely in areas with forest cover [9]. Overall, our study emphasizes the importance of considering seasonal and environmental factors when studying roadkill and identifying hotspots. Such insights can inform mitigation efforts to reduce roads’ impact on wildlife populations. The implementation of various species-specific mitigation measures, alongside general measures like speed limits, warning signs, and wildlife crossings, can help to minimize and mitigate roadkill in national parks and other natural areas.

We acknowledge the limitations of this study. Further research is needed to investigate the factors driving differences in major roadkill species between Banke and Bardia National Parks. Future studies will also explore species-specific patterns by linking kill data with crop seasonality and habitat availability, particularly grasslands and scrublands. Including additional species in the analysis will deepen our understanding of how land use and environmental factors influence wildlife mortality and conflict risk.

## 5. Conclusions

The spatial and temporal analysis of wildlife-vehicle collision (WVC) incidents provides crucial information for future policy decisions and affirmative actions. This study aims to understand WVC to enhance both human safety and ecological balance. Analysis of roadkill in Banke and Bardia National Parks in Nepal showed significant variation over time and location, with Banke recording higher rates, likely due to differences in mitigation, road structure, and habitat quality, as well as there being no buffering area next to the core park. Wild boars and spotted deer, key prey for tigers, were most frequently killed, potentially increasing human-tiger conflict. Seasonal patterns revealed that reptiles were more vulnerable in the wet season, while mammals were at greater risk in summer and winter. Hotspots near water bodies and forests highlight the need for wildlife-friendly infrastructure, strategic habitat management, and public awareness. Most incidents occurred at dawn and dusk, suggesting that targeted interventions during these times are crucial.

To address these challenges, key policy recommendations include developing wildlife-friendly infrastructures such as crossings at identified hot spots and implementing traffic management measures like speed limits and traffic control. Climate resilience strategies should also be integrated into wildlife conservation policies, considering the influence of temperature and precipitation on roadkill patterns. Protecting natural habitats, particularly around water bodies, is essential to reducing wildlife mortality. Continuous monitoring of roadkill hotspots and species-specific mitigation measures will further strengthen conservation efforts. Ongoing research and data collection are necessary to refine these strategies and assess their effectiveness. A broader comparative analysis across multiple landscapes is recommended to enhance the relevance and applicability of the findings. Despite limitations, this case study from Nepal contributes to sustainable conservation planning and supports both local and global policy aimed at achieving the 2030 SDGs and promoting healthy human-wildlife coexistence.

## Figures and Tables

**Table 1:** Summary table for roadkill incidents recorded based on months and years, where months seem to be more important predictors than years.

Term	Estimate	Std. Error	z-Value	*p*-Value	edf	Chi-sq
Intercept	2.02	0.044	45.64	<0.001	-	-
s (Year)	-	-	-	0.369	1.45	1.25
s (Month)	-	-	-	0.008	1.94	7.73

Note: edf stands for the empirical distribution function.

**Table 2:** Test statistics of roadkill with the variables considered in this study.

Coefficients	Estimate	SE	z Value	Pr(>|z|)	CI.Lower	CI.Upper
Intercept	0.4683	0.455	1.029	0.3034	−0.467	1.417
Crop Land (km^2^)	−0.2483	0.1385	−1.793	0.073	−0.537	0.039
Forest (km^2^)	0.2343	0.122	1.92	0.0549	−0.028	0.505
Water Body (km^2^)	1.2214	0.615	1.986	0.0471	0.050	2.712
No. of Turnings	−0.395	0.1662	−2.377	0.0174	−0.729	−0.061

Note: SE stands for standard errors and CI stands for confidence interval. A 5% significance level is considered throughout the study unless otherwise stated.

## Data Availability

Raw data can be provided upon a genuine request to the first author.
